# Effects of low molecular weight heparin on inflammatory, coagulation, and immune markers in hyperlipidemic acute pancreatitis

**DOI:** 10.5937/jomb0-58836

**Published:** 2026-01-28

**Authors:** Yujie Lu, Xinchao Zhu, Caixia Wen, Qiang Zhang, Li Zhao, Jia Ling

**Affiliations:** 1 Department of Gastroenterology, Yancheng Third People's Hospital (The Yancheng School of Clinical Medicine of Nanjing Medical University), Jiangsu, China

**Keywords:** low molecular weight heparin, hyperlipidemic acute pancreatitis, TNF-a, IL-6, IL-8, coagulation, immunoglobulins, amylase, fibrinogen, prothrombin time, immune response, inflammatory markers, heparin niske molekularne mase, hiperlipidemijski akutni pankreatitis, TNF-a, IL-6, IL-8, koagulacija, imunoglobulini, amilaza, fibrinogen, protrombinsko vreme, imunološki odgovor, inflamatorni markeri

## Abstract

**Background:**

This study investigates the biochemical impact of low molecular weight heparin (LMWH) on key immunological, coagulation, and inflammatory markers in patients with hyperlipidemic acute pancreatitis (HLAP). The objective is to elucidate the role of LMWH in modulating serum immunoglobulins (IgA, IgG, IgM), coagulation parameters (PT, TT, FIB, APTT), proinflammatory cytokines (TNF-a, IL-6, IL-8), and digestive enzyme activity (amylase), thereby providing insight into its therapeutic mechanism.

**Methods:**

A total of 100 HLAP patients treated between January 2022 and December 2024 were assigned to a control group (CG) receiving standard medical treatment, and an experimental group (EG) receiving standard treatment plus LMWH. Biomolecular markers were analysed to assess changes in coagulation dynamics, inflammatory signalling, immunoglobulin response, and lipid metabolism. Comparative analysis between groups was conducted to evaluate the biochemical effects of LMWH.

**Results:**

Compared to the control group, the LMWH-treated group demonstrated significant reductions in inflammatory mediators: TNF-a decreased by 38.2%, IL-6 by 34.5%, and IL-8 by 36.7% (all P&lt;0.01). Serum amylase and urinary amylase levels declined by 41.3% and 39.6%, respectively (P&lt;0.01). Coagulation profiles improved with PT prolonged by 13.8%, TT by 15.2%, FIB reduced by 12.4%, and APTT normalised by a 17.1% increase (P&lt;0.01). Immune markers IgA, IgG, and IgM increased by 22.5%, 26.3%, and 24.8%, respectively (P&lt;0.01). Additionally, the LMWH group showed better lipid regulation (TG reduced by 45.7%) and a lower complication rate (6% vs. 22%, P=0.02).

**Conclusions:**

LMWH exhibits a multifaceted biochemical effect in HLAP patients, encompassing anti-inflammatory action, immunomodulation, and correction of coagulation abnormalities. These findings support LMWH as a potential adjunctive therapeutic agent in the biochemical management of HLAP warranting further molecular studies to explore its mechanistic pathways.

## Abstract (sr)

**Uvod:**

Ova studija ispituje biohemijski uticaj heparina niske molekularne mase (HNMM) na ključne imunološke, koagulacione i inflamatorne markere kod pacijenata sa hiperlipidemijskim akutnim pankreatitisom (HLAP). Cilj je da se razjasni uloga HNMM u modulaciji serumskih imunoglobulina (IgA, IgG, IgM), koagulacionih parametara (PT, TT, FIB, APTT), proinflamatornih citokina (TNF-a, IL-6, iL-8) i aktivnosti digestivnih enzima (amilaza), čime se pruža uvid u njegov terapijski mehanizam.

**Metode:**

Ukupno 100 pacijenata sa HLAP lečenih između januara 2022. i decembra 2024. godine bilo je raspoređeno u kontrolnu grupu (KG), koja je dobijala standardno medicinsko lečenje, i eksperimentalnu grupu (EG), koja je pored standardnog lečenja primala i HNMM. Biomolekularni markeri su analizirani radi procene promena u koagulacionoj dinamici, inflamatornoj signalizaciji, imunoglobulinskom odgovoru i metabolizmu lipida. Sprovedena je komparativna analiza između grupa sprovedena kako bi se procenili biohemijski efekti HNMM.

**Rezultati:**

U poređenju sa kontrolnom grupom, grupa koja je primala HNMM je pokazala značajno smanjenje inflamatornih medijatora: TNF-a je smanjen za 38,2%, IL-6 za 34,5%, a IL-8 za 36,7% (svi P&lt;0,01). Nivoi serumskih i urinarnih amilaza opali su za 41,3%, odnosno 39,6% (P&lt;0,01). Koagulacioni profil se poboljšao produženjem PT za 13,8%, TT za 15,2%, smanjenjem FIB za 12,4% i normalizacijom APTT porastom od 17,1% (P&lt;0,01). Imunološki markeri IgA, IgG i IgM povecani su za 22,5%, 26,3% i 24,8%, redom (P&lt;0,01). Pored toga, grupa koja je primala HNMM pokazala je bolju regulaciju lipida (TG smanjen za 45,7%) i nižu stopu komplikacija (6% naspram 22%, P=0,02).

**Zaključak:**

HNMM pokazuje višestruke biohemijske efekte kod pacijenata sa HLAP uključujući antiinflamatorno delovanje, imunomodulaciju i korekciju koagulacionih poremećaja. Ovi nalazi podržavaju upotrebu HNMM kao potencijalnog pomoćnog terapijskog agensa u biohemijskom upravljanju HLAP-om, a potrebna su dalja molekularna istraživanja radi razjašnjavanja njegovih mehanizama delovanja.

## Introduction

Hyperlipidemic acute pancreatitis (HLAP) is a subtype of acute pancreatitis characterised by significantly elevated serum triglyceride (TG) levels, often exceeding 11.3 mmol/L [1] [2] [3] [4] [5]. This condition is increasingly prevalent due to the rising incidence of metabolic syndromes such as obesity, type 2 diabetes, and primary hyperlipidemia [6] [7]. Biochemically, HLAP arises when high concentrations of triglyceride-rich lipoproteins are hydrolysed by pancreatic lipase, leading to an excessive release of free fatty acids (FFAs) [8] [9] [10]. These FFAs, in their unbound form, are cytotoxic to pancreatic acinar cells and capillary endothelial cells, initiating a cascade of cellular damage, oxidative stress, and systemic inflammation [11] [12] [13].

The early phase of HLAP is dominated by local pancreatic inflammation and enzyme activation. At the same time, the later stages involve systemic inflammatory response syndrome (SIRS) and potentially multiple organ dysfunction syndrome (MODS) [14]. From a molecular perspective, these processes are driven by the overexpression of proinflammatory cytokines such as tumour necrosis factor-alpha (TNF-α), interleukin-6 (IL-6), and interleukin-8 (lL-8) [15] [16]. These cytokines amplify the immune response by recruiting neutrophils, activating monocytes, and enhancing the production of reactive oxygen species (ROS), all of which further contribute to acinar necrosis and vascular injury [17] [18].

In parallel, HLAP exerts significant disruptions in coagulation homeostasis. The inflammatory milieu promotes endothelial injury and activation of the coagulation cascade, leading to measurable changes in biomarkers, including prolonged prothrombin time (PT), altered thrombin time (TT), dysregulated fibrinogen (FIB) levels, and abnormal activated partial thromboplastin time (APTT) [19] [20]. This hypercoagulable state may exacerbate pancreatic ischemia by promoting microvascular thrombosis and impairing oxygen delivery to inflamed tissues [21].

Immune dysfunction also plays a critical role in HLAP pathophysiology. Patients often exhibit decreased levels of immunoglobulins - IgA, IgG, and IgM - reflecting impaired humoral immune responses. These reductions correlate with higher risks of bacterial translocation from the gut, systemic infections, and sepsis [22]. Moreover, HLAP patients typically present with elevated pancreatic enzymes, including serum and urinary amylase, indicative of ongoing acinar cell damage and enzymatic leakage into the circulation.

Low-molecular-weight heparin (LMWH), a widely used anticoagulant, has demonstrated potential beyond its classical role in preventing thromboembolism. Mechanistically, LMWH acts by potentiating antithrombin III activity, thereby inhibiting factor Xa and thrombin formation. Importantly, it also exerts pleiotropic biochemical effects relevant to HLAP

It attenuates cytokine-mediated inflammation by suppressing neutrophil adhesion and migration. It improves pancreatic microcirculation, reducing tissue hypoxia. It may enhance lipoprotein lipase (LPL) activity, facilitating the hydrolysis and clearance of chylomicrons and very low-density lipoproteins (VLDL), thereby lowering serum TG levels. It contributes to immunomodulation by potentially stabilising or enhancing immunoglobulin levels.

In light of these multifaceted biochemical effects, LMWH represents a promising agent in modifying the pathological processes of HLAP at the molecular level. However, the current body of research lacks comprehensive studies evaluating its impact on key biochemical markers associated with HLAP progression and resolution [23] [24].

This study aims to fill that gap by systematically investigating the effects of LMWH on a panel of clinically relevant molecular biomarkers in HLAP patients. Specifically, we examine changes in inflammatory cytokines (TNF-α, IL-6, IL-8), immunoglobulins (IgA, IgG, IgM), coagulation indicators (PT, TT, FIB, APTT), and pancreatic enzymatic activity (serum and urinary amylase). Through this biochemical lens, we seek to elucidate the mechanistic role of LMWH in modulating disease severity and supporting systemic recovery in HLAP

## Materials and methods

### Study design and participants

This prospective controlled study was conducted on 100 patients diagnosed with hyperlipidemic acute pancreatitis (HLAP) who were treated at our tertiary care centre between January 2022 and December 2024. Eligible participants were randomly allocated into two equal groups: a control group (CG, *n*=50) receiving standard treatment and an experimental group (EG, *n* = 50) receiving standard treatment plus low molecular weight heparin (LMWH).

Randomisation was performed using a computer-generated random number sequence, and block randomisation with a block size of 4 was used to ensure balanced group sizes throughout the study. Allocation concealment was maintained by using sealed, opaque, sequentially numbered envelopes prepared by an independent statistician not involved in participant recruitment or treatment administration.

Sample size calculation was based on preliminary data suggesting a mean 25% reduction in TNF-α levels with LMWH therapy. Assuming a significance level of α = 0.05 and 80% power to detect a between-group difference of 20% in inflammatory marker reduction, a minimum of 45 patients per group was required. To account for potential dropouts, the sample size was increased to 50 per group.

Inclusion criteria comprised a confirmed diagnosis of acute pancreatitis based on revised Atlanta classification criteria, with serum triglyceride levels 11.3 mmol/L, or levels between 5.65 and 11.3 mmol/L accompanied by chylous serum appearance. Exclusion criteria included pancreatitis due to other causes (e.g., biliary, alcohol-related), chronic comorbidities (such as cardiovascular disease or malignancies), psychiatric disorders, recent anticoagulant use, or known contraindications to LMWH therapy. All participants provided written informed consent prior to inclusion, and the institutional medical ethics committee approved the study protocol.

### Intervention protocol

**Figure 1:**
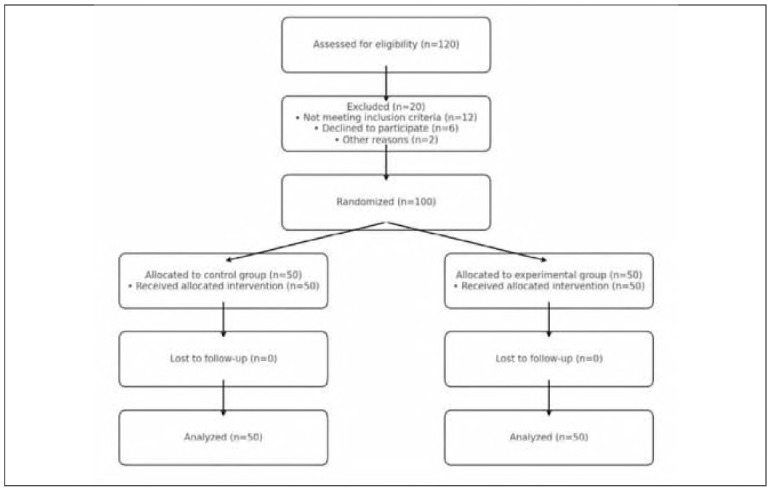
CONSORT flow diagram of study design.

All patients received conventional biochemical stabilisation for HLAP, including fasting, fluid replacement, electrolyte correction, inhibition of pancreatic enzyme secretion, and triglyceride-lowering interventions. In addition to these treatments, patients in the experimental group received subcutaneous injections of low-molecular-weight heparin (Enoxaparin sodium, 5000 IU/0.2 mL; Jilin Huakang Pharmaceutical Co., China), administered twice daily for seven consecutive days. The LMWH dosing regimen (enoxaparin sodium 5000 IU, administered twice daily for 7 days) was selected based on its established pharmacokinetics and prior use in patients with moderate to severe acute pancreatitis [25] [26]. Renal function was assessed prior to LMWH initiation, and patients with creatinine clearance <30 mL/min were excluded (or dose-adjusted if applicable). A fixed dose was used irrespective of body weight due to the short treatment duration and biochemical focus of the study. This dose falls within the commonly used 4000-6000 IU range, which has been shown to be effective for anti-inflammatory and anticoagulant effects without increasing the risk of bleeding. Enoxaparin also enhances lipoprotein lipase activity, supporting triglyceride clearance, and improves microcirculation by inhibiting factor Xa and thrombin. The twice-daily schedule ensures stable plasma levels during the acute phase of HLAP [27] [28]. Figure 1

No additional nutritional interventions or clinical care measures were evaluated in this biochemical-focused study.

### Sample collection and processing

For biochemical analyses, fasting peripheral venous blood samples (5 mL) were collected from each patient at two time points: immediately prior to treatment initiation (baseline) and on the seventh day following the intervention. Blood was collected using sterile serum separation tubes, allowed to clot for 30 minutes at room temperature, and centrifuged at 3000 rpm for 10 minutes at 4°C. The supernatant serum was aliquoted into sterile cryovials and stored at -80°C until laboratory evaluation.

### Biochemical analyses

Serum and urine samples were analysed for a panel of biochemical markers associated with pancreatic function, lipid metabolism, immune response, inflammatory status, coagulation, and nutritional condition.

Serum and urinary amylase levels were measured by enzymatic colourimetric methods using the Beckman Coulter AU5800 Chemistry Analyzer (USA), with commercial reagents obtained from Beckman Coulter Diagnostics (Cat. No. OSR6179 for serum amylase and OSR6284 for urinary amylase). Lipid profiles - including triglycerides (TG), total cholesterol (TC), low-density lipoprotein cholesterol (LDL-C), and high-density lipoprotein cholesterol (HDL-C) - were assessed using enzymatic assays based on glycerol-3-phosphate oxidase and cholesterol esterase methods. These analyses were performed on the Hitachi 7600 automatic biochemical analyser (Japan) using reagent kits from Sekisui Medical (Cat. No. 442-00601 for TG and 442-00611 for TC).

Inflammatory markers, including tumour necrosis factor-alpha (TNF-α), interleukin-6 (IL-6), interleukin-8 (IL-8), and C-reactive protein (CRP), were quantified using enzyme-linked immunosorbent assay (ELISA). TNF-α was measured with R&D Systems kit (Cat. No. DTA00C), IL-6 with R&D Systems kit (Cat. No. D6050), IL-8 with R&D Systems kit (Cat. No. D8000C), and CRP with BioVendor kit (Cat. No. RD191027100). All ELISA assays were run in duplicate, and absorbance was read at 450 nm with correction at 570 nm using a BioTek ELx800 Microplate Reader (USA).

Coagulation parameters - including prothrombin time (PT), thrombin time (TT), activated partial thromboplastin time (APTT), and fibrinogen (FIB) - were evaluated using a Sysmex CS-5100 automated coagulation analyser (Japan). Reagents included Siemens Thromborel S for PT, Pathromtin SL for APTT, Siemens Thrombin Reagent for TT, and Multifibren U for fibrinogen quantification.

Nutritional status was assessed by measuring total protein (TP), albumin (ALB), and transferrin (TRF) using bromocresol green (BCG) and turbidi-metric assays on the Beckman Coulter AU5800 platform. The respective reagents used were Beckman Coulter kits (Cat. No. OSR6214 for TP and OSR6102 for ALB). Immunoglobulin levels - including IgA, IgG, and IgM - were determined using immunoturbidimetric methods with the Roche Cobas c702 analyser. Commercial kits from Roche/Hitachi were used: IgA (Cat. No. 05172069 190), IgG (Cat. No. 05172034 190), and IgM (Cat. No. 05172114 190).

### Outcome measures

The primary outcomes of this study were the biochemical changes in inflammatory cytokines (TNF-α, IL-6, IL-8, CRP), immunoglobulin levels (IgA, IgG, IgM), coagulation indices (PT, TT, FIB, APTT), serum and urinary amylase activity, lipid parameters (TG, TC, LDL-C, HDL-C), and nutritional biomarkers (TP, ALB, TRF), measured before and after the 7-day treatment period. These indicators were chosen to comprehensively evaluate the systemic biochemical effects of LMWH in HLAP patients.

### Statistical analysis

All data were analysed using SPSS version 26.0 (IBM Corp., Armonk, NY, USA). Continuous variables were expressed as mean ± standard deviation (SD). Comparisons of pre- and post-treatment values within each group were made using paired Student's t-tests, while intergroup differences were analysed using independent samples t-tests. Categorical data were compared using chi-square (χ^2^) tests. A two-tailed p-value <0.05 was considered statistically significant. Data visualisation and biomarker trend analysis were performed using GraphPad Prism 9.0 (GraphPad Software, USA).

## Results

### Total effective rate in both groups

Seven days after treatment, the total effective rate of the experimental group (EG) was 96.00%, significantly higher than that of the control group (CG) at 82.00% (*P*=0.03). Pairwise comparisons showed that the EG had substantially fewer »Ineffective« cases (*P*=0.04) and a trend toward more »Obviously effective« responses compared to the CG (*P*=0.28), although the difference was not statistically significant.

### Disappearance time of abdominal distension and abdominal pain, exhaustion recovery time, defecation recovery time, bowel sound recovery time and length of hospitalisation in both groups

**Figure 2:**
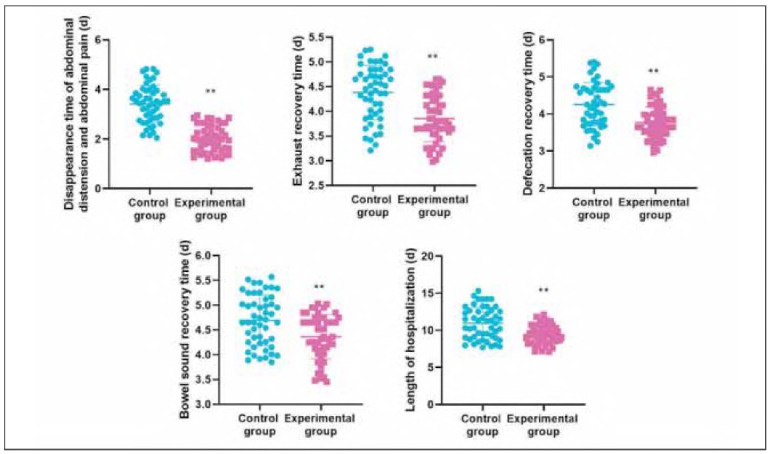
Disappearance time of abdominal distension and abdominal pain, exhaust recovery time, defecation recovery time, bowel sound recovery time and length of hospitalisation in both groups. **P < 0.01

Relative to the CG, the EG had shorter disappearance time of abdominal distension and abdominal pain, exhaust recovery time, defecation recovery time, bowel sound recovery time and length of hospitalisation (*P*<0.01, Figure 2). Table 1

**Table 1:** Total effective rate in both groups.

Group	Cases	Obviously<br>effective	Effective	Ineffective	Total effective<br>rate	χ^2^	P
Control group	50	24 (48.00%)	17 (34.00%)	9 (18.00%)	41 (82.00%)	5.01	0.03
Experimental group	50	28 (56.00%)	20 (40.00%)	2 (4.00%)	48 (96.00%)		
Pairwise P-values		0.28	0.48	**0.04**			

### Serum amylase and urine amylase levels in both groups

**Figure 3:**
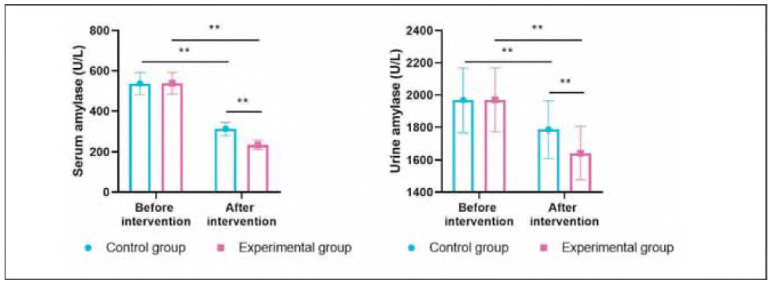
Serum amylase and urine amylase levels in both groups. **P < 0.01

Before intervention, serum amylase and urine amylase levels showed no difference between the 2 groups (P>0.05). After intervention, serum amylase and urine amylase levels were diminished in 2 groups (P<0.05). Importantly, relative to the CG, the EG had lower serum amylase and urine amylase levels (P<0.05, Figure 3).

### Blood lipid levels in both groups

**Figure 4:**
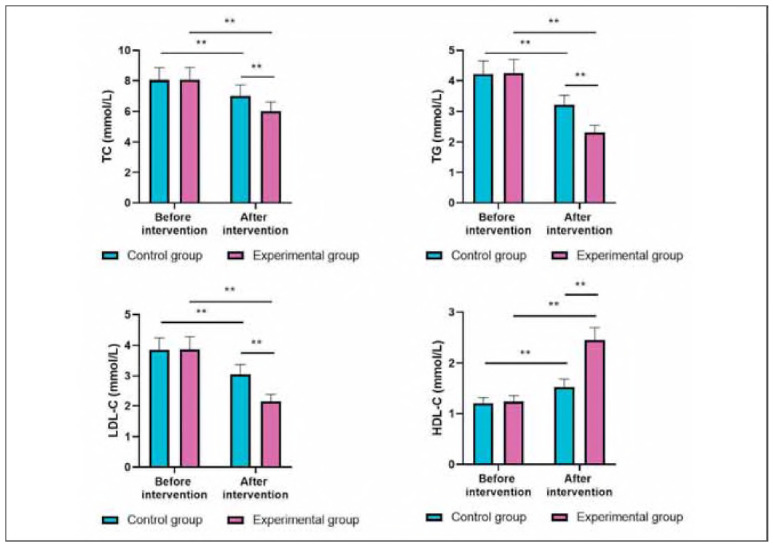
Blood lipid levels in both groups. **P < 0.01

Before intervention, the levels of TC, TG, LDL-C, and HDL-C showed no difference between the two groups (P>0.05). After intervention, the levels of TC, TG and LDL-C were diminished while HDL-C levels were elevated in 2 groups (P<0.05). Importantly, relative to the CG, the EG had better improvements of the blood lipid markers (P<0.05, Figure 4).

### Coagulation function in both groups

**Figure 5:**
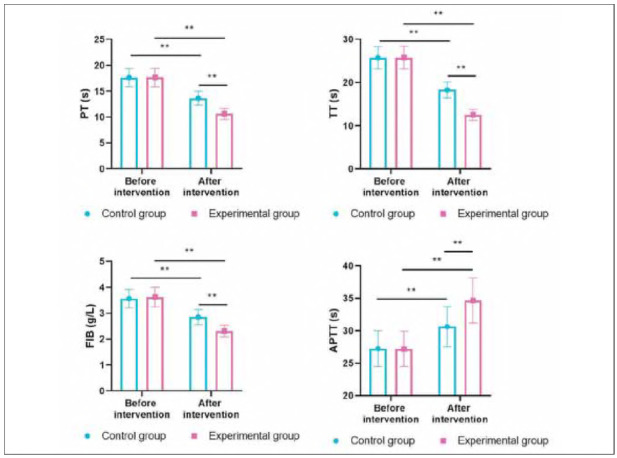
Coagulation function in both groups. **P < 0.01

Before intervention, the levels of PT, TT, FIB, and APTT showed no difference between the two groups (P>0.05). After intervention, the levels of PT, TT, and FIB were decreased, while APTT levels were elevated in both groups (P<0.05). Importantly, relative to the CG, the EG had better improvements of the above coagulation function indexes (P<0.05, Figure 5).

In the experimental group, APTT increased from 30.2±3.1 seconds to 35.4±3.3 seconds (Δ= + 5.2 seconds, P<0.01), representing a 17.2% rise post-treatment. PT and TT also showed moderate prolongations (13.8% and 15.2%, respectively), while fibrinogen levels decreased by 12.4%. These changes reflect a measurable anticoagulant effect but remain within therapeutic and physiologically acceptable limits. Notably, no patients in the LMWH group experienced major bleeding events or required transfusion, suggesting that the observed laboratory changes were not associated with increased bleeding risk. This highlights the clinical safety of the fixed-dose LMWH regimen over the short-term treatment window used in this study.

### Levels of inflammatory markers in both groups

**Figure 6:**
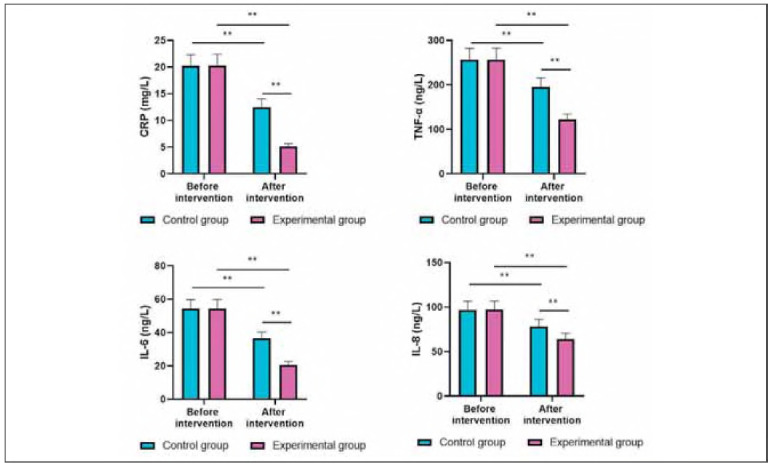
Levels of inflammatory markers in both groups. **P < 0.01

Before intervention, the levels of CRP TNF-α, IL-6, and IL-8 showed no difference between the two groups (P>0.05). After intervention, the levels of the above inflammatory markers were diminished in 2 groups (P<0.05). Importantly, relative to the CG, the EG had lower levels of the above inflammatory markers (P<0.05, Figure 6).

### Nutritional indexes and immune indexes in both groups

**Figure 7:**
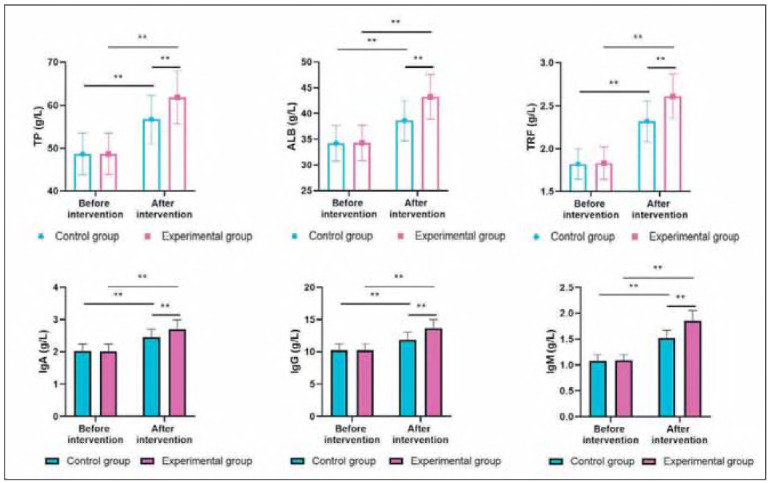
Nutritional indexes and immune indexes in both groups. **P < 0.01

Before intervention, the levels of TP, ALB, and TRF showed no difference between the two groups (P>0.05). Following the intervention, the levels of the aforementioned nutritional indices increased in both groups (P<0.05). Importantly, relative to the CG, the EG had higher levels of the above dietary indexes (P<0.05, Figure 7). Before intervention, the levels of IgA, IgG, and IgM showed no difference between the two groups (P>0.05). After intervention, the levels of the above immune indexes were elevated in 2 groups (P<0.05). Importantly, relative to the CG, the EG had higher levels of the above immune indexes (P<0.05, Figure 7).

### Incidence of complications in both groups

Compared to the CG, the EG had a lower incidence of complications (P<0.05, Table 2).

**Table 2:** Incidence of complications in both groups.

Groups	Cases	Gastrointestinal<br>bleeding	Rash	Multiple organ<br>dysfunction syndrome	Total incidence rate
Control group	50	3 (6.00)	3 (6.00)	5 (10.00)	11 (22.00)
Experimental group	50	1 (2.00)	1 (2.00)	1 (2.00)	3 (6.00)
χ^2^					5.32
P					0.02

## Discussion

HLAP is caused by increased TG levels in the blood, leading to pancreatic microcirculation disorders and pancreatic injury diseases [29]. Currently, the known pathogenesis of HLAP primarily involves free fatty acid injury, pancreatic microcirculation disorder, inflammatory response, endoplasmic reticulum stress, calcium overload, genetic factors, and gene mutation [30]. Therefore, the rapid reduction of TG levels, clearance of inflammatory mediators, and elimination of the inflammatory response are key to the treatment of HLAP [31].

HLAP treatment includes fasting, fluid supplementation, acid inhibition, enzyme inhibition and other basic symptomatic supportive treatment [32]. The most important treatment measure is to reduce the TG level rapidly. The main pharmacological effect of heparin is anticoagulation, which inhibits the action of coagulation factors IIa (thrombin), Xa and IXa to produce an anticoagulation impact [33]. LMWH is a preparation of heparin, which has a more decisive action and better safety, and its main anticoagulation mechanisms include inhibiting the action of thrombin and inhibiting the effect of coagulation active factor Xa [34]. Therefore, LMWH can treat pancreatic microcirculation disorder, prevent the formation of microthrombosis, stabilise pancreatic hemodynamics, and improve ischemia-reperfusion injury of pancreatic tissue [35]. Meanwhile, LMWH can inhibit the release of various inflammatory cytokines through the complement system, thereby reducing inflammation by preventing the accumulation of white blood cells and blocking the release of inflammatory factors [36]. In addition, LMWH plays a vital role in stimulating capillary endothelial cells to release lipoprotein lipase into the blood, which can decompose TG into fatty acids and glycerides for tissue oxidation, energy supply and storage, thereby reducing the serum TG level to treat HALP [37].

Nutritional support is a crucial component of the treatment strategy for patients with acute pancreatitis, ensuring adequate nutrient intake and maintaining the balance of body energy metabolism [38]. Parenteral nutrition support can shorten the recovery time of patients without affecting pancreatic secretion. Still, this intervention method does not conform to the normal physiological function of the human pancreas. As a result, with the extension of the intervention time, some patients may experience adverse conditions, such as diarrhoea, affecting gastrointestinal function [39]. Studies have found that early nutritional support can promote the recovery of gastrointestinal function, improve serum indicators, and accelerate the rehabilitation process [40].

The results of our study indicated that relative to the CG, the EG had higher total effective rate, shorter disappearance time of abdominal distension and abdominal pain, exhaust recovery time, defecation recovery time, bowel sound recovery time and length of hospitalisation and lower serum amylase and urine amylase levels, indicating that LMWH combined with early enteral nutrition patient care was effective in promoting physical recovery of HLAP patients. The reason can be seen that LMWH can stimulate the release of fibrinolytic substances in the vascular endothelia of the body, improve its activity, and then inhibit platelet aggregation, so that the body's blood hypercoagulable state can be improved, as an auxiliary treatment of conventional therapy, can improve the symptoms of patients while improving the therapeutic effect [41]. Meanwhile, as a nutrient, enteral nutrient solution can stimulate the secretion of digestive fluids, gastrointestinal hormones, enzymes, and immune substances, promote gastrointestinal peristalsis, and more closely align with normal physiological functions, thereby facilitating the recovery of gastrointestinal function and the recovery from diseases [42]. Additionally, early enteral nutrition can better meet the physiological needs of patients with severe acute pancreatitis and has a significant effect on promoting protein synthesis and correcting the stress state, thereby effectively promoting the recovery of serum amylase and urine amylase levels [43].

Our study indicated that after intervention, relative to the CG, the EG had better improvements of blood lipid markers, better improvements of coagulation function indexes, and lower levels of inflammatory markers, suggesting that LMWH combined with early enteral nutrition care could improve blood lipid level, coagulation function and inflammatory response in HLAP patients. The reasons may be as follows: LMWH, as an anticoagulant, can inhibit the expression of proinflammatory factors caused by endotoxin, thus hindering the synthesis of inflammatory mediators [44]. LMWH can reduce the damage to vascular endothelial cells and promote the release of tissue plasminogen activator, thereby reducing blood viscosity and improving microcirculation [45]. LMWH can promote the rapid release of tissue factors, accelerate the increase in lipoprotein lipase activity, reduce tissue plasminogen activator, regulate cell adhesion molecules, and accelerate the degradation of chylomicrons, thereby promoting the decline of TG, enhancing the lipid-lowering effect, and accelerating the disease outcome [46]. In addition, early enteral nutrition can accelerate gastrointestinal motility, protect the function of the small intestine and mucosal structure, and promote the secretion of gastrointestinal hormones and gastric acid, thereby inhibiting the inflammatory response [47].

While LMWH significantly influenced coagulation parameters (prolonged PT and TT, normalised APTT, and improved FIB levels), these changes remained within accepted therapeutic ranges. They were not associated with clinically significant bleeding events. The absence of major hemorrhagic complications in the experimental group suggests that the anticoagulant effect of LMWH at the administered dose was well tolerated. Nevertheless, given the prothrombotic and hypocoagulable risks in HLAP ongoing monitoring of coagulation indices is essential. Future studies should further assess the safety profile of LMWH, particularly in patients with borderline coagulation status or concurrent risk factors for bleeding.

Although significant reductions in TNF-α, IL-6, and IL-8 levels were observed following LMWH administration, the clinical relevance of these changes remains uncertain. These proinflammatory cytokines are strongly associated with disease severity and progression in acute pancreatitis. However, the present study did not incorporate standardised severity scoring systems such as APACHE II or BISAP to correlate biochemical improvements with clinical outcomes. Future studies should include such assessments to better clarify whether reductions in inflammatory markers translate into meaningful improvements in disease severity or prognosis.

Our study also showed that after intervention, relative to the CG, the EG had higher levels of nutritional indexes, higher levels of immune indexes and lower incidence of complications, implying that LMWH combined with early enteral nutrition care could enhance the nutritional status and immune function, as well as reduce the incidence of complications in HLAP patients. The reason is that the implementation of early enteral nutrition care can provide timely supplementation of various nutrients required by the body, protect the gastrointestinal mucosa and tissue function to the maximum extent, promote the reversal of gastrointestinal mucosal damage, enhance gastrointestinal immune function, and thus improve the nutritional status of patients [48]. In addition, early enteral nutrition can be directly absorbed through the intestine, enhance the function of the gastrointestinal mucosal barrier, and promote the direct entry of nutritional factors into the liver, thereby playing a leading role in treatment and improving the nutritional status and immune function of the body [49]. The treatment of LMWH can improve the microcirculation of the pancreas and other organs, providing favourable conditions for the absorption and dissipation of inflammation. It promotes the faster elimination of symptoms, such as abdominal pain and abdominal distension, and reduces the occurrence of complications in patients [50].

This study has several significant limitations that should be considered when interpreting the results. First, the study was conducted at a single tertiary care centre, which may limit the generalizability of the findings to broader clinical settings. Second, the follow-up duration was limited to seven days, which prevented the assessment of long-term outcomes, such as recurrence, sustained immunological changes, or late-onset complications. Third, the study did not employ validated clinical severity scoring systems (e.g., APACHE II, BISAP), which limits the ability to correlate biochemical improvements with clinical severity or prognosis. Additionally, LMWH was administered at a fixed dose without adjustments for patient weight or renal function, despite known pharmacokinetic variability. These factors may influence both efficacy and safety, particularly in diverse patient populations. Future multicenter, randomised studies with extended follow-up and individualised dosing protocols are necessary to validate and expand upon these findings.

In conclusion, LMWH combined with early enteral nutrition care is an effective management strategy for HLAP patients, promoting physical recovery, improving coagulation function and inflammatory response, enhancing nutritional status and immune function, and reducing the incidence of complications.

## Dodatak

### Acknowledgements

None.

### Funding

There is no funding to report.

### Ethics approval and consent to participate

This study was approved by the Ethics Committee of Yancheng Third People's Hospital (The Yancheng School of Clinical Medicine of Nanjing Medical University). All subjects signed informed consent.

### Availability of data and materials

The datasets generated during and/or analysed during the current study are available from the corresponding author upon reasonable request.

### Authors' contribution

Yujie Lu and Caixia Wen contributed equally to this paper.

### Conflict of interest statement

All the authors declare that they have no conflict of interest in this work.
